# Laryngeal vocal and endoscopic alterations after thyroidectomy under local anesthesia and hypnosedation

**DOI:** 10.1016/S1808-8694(15)30489-4

**Published:** 2015-10-19

**Authors:** Lincoln Santos Souza, Agrício Nubiato Crespo, Jovany Luís Alves de Medeiros

**Affiliations:** 1 Full Member of the Brazilian College of Surgeons TCBC – CCP -PB. MSc on Medical Sciences – Campinas State University – UNICAMP – SP Head and Neck Surgeon at the Federal University of Campina Grande UFCG – PB; Head of the Head and Neck Surgery Service at FAP Hospital – Campina Grande-PB; 2 Head of the ENT Department at the Medical School of the Campinas State University – UNICAMP – Campinas – SP; 3 Neurology and Neurophysiology at Paraíba State University – UEPB – Campina Grande – PB

**Keywords:** dysphonia, thyroidectomy

## Abstract

Vocal alterations after thyroidectomy are generally related to laryngeal nerve injury or laryngotracheal mobility disorders caused by postoperative fibrosis or strap muscle lesion.

**Aim:**

this study aims to evaluate the frequency of vocal and rima glottidis disorders after thyroidectomy.

**Materials and method:**

This is a prospective study based on 35 patients submitted to thyroidectomy under local anesthesia and hypnosedation. All patients underwent voice auditory perception evaluation, voice acoustic tests and videolaryngostroboscopy preoperatively, and at one week and at 30 days postoperatively. Bilateral cricothyroid muscle electromyography was performed on the thirtieth day after surgery to confirm the presence of injury in the external branch of the superior laryngeal nerve.

**Results:**

14.3% of the patients presented posterior glottis deviation before surgery and normal electromyography findings. Transient and permanent vocal alteration occurred in 25.7% and 14.2% of the patients respectively.

**Conclusion:**

voice disorders evaluated after voice auditory perceptive evaluation and voice acoustic tests were more intense in the group with superior laryngeal nerve external branch injury than in the injury-free dysphonic patient group. Oblique glottis can be present in normal patients; however its onset after thyroidectomy is indicative of superior laryngeal nerve external branch lesion.

## INTRODUCTION

Thyroidectomy is currently the most frequently performed endocrine procedure. Surgical treatment of the diseased thyroid gland has developed quite significantly within the past years, as it has become a multidisciplinary effort. New technologies to improve hemostasis and perioperative monitoring of the laryngeal nerves have turned thyroid surgery into a safer, less fraught with complication procedure. There is growing interest in thyroid surgery done in combination with local or regional anesthesia, sedation and analgesia, increasing the number of procedures performed in an outpatient setting^1^, ^2^, ^3^. Additionally, thyroidectomy procedures have been increasingly performed by physicians trained at specialized head and neck surgery centers. All technological and professional progress has improved the quality of the procedures performed, resulting in fewer postoperative complications. Voice disorders resulting from laryngeal nerve injury secondary to thyroidectomy may significantly impair the lives of patients, more so of those who use their voices professionally. Injuries to the recurrent laryngeal nerve are easily diagnosed immediately after surgery through direct observation of paralyzed vocal folds. Differently, injuries to the external branch of the superior laryngeal nerve may only be observed when the patient vocalizes higher pitch sounds. As many as 87% of patients may experience voice disorders immediately after surgery diagnosed through voice auditory perception evaluation and voice acoustic tests, even in patients with uninjured recurrent laryngeal nerve4. The main clinical characteristics of voice disorders are vocal fatigue and hoarseness. Therefore, it is important to assess vocal and laryngeal condition after thyroidectomy under local anesthesia and hypnosedation in order to test vocal control perioperatively through phonation during manipulation of the laryngeal nerves.

## OBJECTIVES

This study aims to assess the prevalence of voice and rima glottidis disorders after thyroidectomy at one week and at thirty days postoperatively.

## MATERIALS AND METHODS

Thirty-five patients (3 males and 32 females) submitted to thyroidectomy between January and May of 2005 under local anesthesia and hypnosedation were assessed. Patient age ranged between 22 and 77 years (average: 43.5). Eligibility criteria included patients submitted to lobectomy accompanied or not by isthmectomy, total thyroidectomy with or without bilateral lymph node resection, done under local anesthesia and hypnosedation on patients with at least 18 years of age. Patients with clinically diagnosed voice disorders, previous neck surgery, neck trauma, and not meeting the above mentioned criteria were excluded. All patients were submitted to a 4-step evaluation process:

1. Subjective voice analysis using the GRBAS scale and measurement of maximum phonation time before surgery, one week after the procedure, and thirty days after surgery. Subjective voice analysis was carried out by two specialized speech therapists using the GRBAS modified scale, in which parameters degree of voice disorder, roughness, breathiness, asthenia, and stress. Maximum phonation time was measured after three sustained emissions of vowel 'a' (as it sounds in Portuguese), the longest being chosen.
2. Voice acoustic analysis before surgery, one week after the procedure, and thirty days after the procedure. The following parameters were assessed: fundamental frequency, percent jitter, percent shimmer, and noise harmonic ratio. Acoustic analysis was conducted in a voice lab using software MDVP – Multidimensional Voice Program – Kay Elemetrics Corporation. Patients were asked to emit and sustain vowel 'a'(as it sounds in Portuguese) in their usual volume and intensity for more than three seconds, with the microphone positioned 15 cm away from the mouth to measure the parameters mentioned above.
3. Videolaryngostroboscopy preoperatively and postoperatively one week and then thirty days after surgery. Videolaryngostroboscopy was performed using an ATMOS device equipped with 70° laryngeal scope after oropharyngeal topic anesthesia using lidocaine at 10%. Patients were asked to emit and sustain vowel 'i'(as it sounds in Portuguese) in their usual intensity, followed by 'i'(as it sounds in Portuguese) emitted in the highest pitch possible to better assess the cricothyroid muscle.
4. Bilateral electromyography of the cricothyroid muscle between days 21 and 30 after surgery. Patients with injured superior laryngeal nerve external branch as seen under electromyography were submitted to electromyography again three months after surgery. Cricothyroid muscle puncture was performed by a head and neck surgeon and electromyographic tracings recorded and interpreted by an electroneurophysiologist. A Compass Portabook II Nicolet electromyograph integrated to a Compaq computer was used.

### Anesthesia and surgery

All patients were operated on by the same surgeon according to the following protocol:

1. Hypnosedation - initiated with midazolam at 0.2mg/kg of bodily weight, 15 mg orally on average forty minutes before surgery. Next, clonidine hydrochloride at 2 – 4 mcg/kg in continuous infusion 10 minutes before surgery.
2. Before local anesthesia injection, patients were given fentanyl or alfentanil intravenously at 2mcg/kg or 15mcg/kg, respectively.
3. Patients were then given a local injection of between 15 and 20 ml of 2% lidocaine with adrenalin in a 1:200,000 dilution rate. The injection was administered along the line of the surgical incision, bilaterally on the medial border of the sternocleidomastoid muscle superiorly on the vicinity of the cricoid cartilage, and inferiorly in the sternal furcula. Cervical plexus blocking was not performed. All patients underwent partial (lobectomy accompanied or not by isthmectomy) or total thyroidectomy with or without bilateral lymph node resection.
4. The conventional surgical approach was followed, with lateral traction of the sternothyroid and sternohyoid muscles after a midline incision was made to prevent them from being detached or sectioned. The cricothyroid space was dissected in all patients to expose the thyroid superior vascular pedicle, composed by the superior thyroid artery and the superior thyroid vein, each carefully approached and ligated separately. None of the patients had the external branch of the superior laryngeal nerve dissected, unless the nerve was naturally exposed after cricothyroid space dissection. The recurrent laryngeal nerve was dissected in all patients after ligation of the middle thyroid vein and the inferior thyroid artery. Patients were then asked to speak so the integrity of the recurrent laryngeal nerve could be assessed. The remainder of the surgery followed the already known steps described in the traditional technique.

### Statistical analysis

Descriptive analysis was performed based on the measurements of position and scatter for continuous variables and frequency tables for categorical variables. ANOVA was used to assess parameter evolution along time for repeated measures with transformation by posts. Differences were identified through contrast profile tests. Statistical tests adopted a 5% significance threshold.

## RESULTS

Patient age ranged between 22 and 77 years (median = 42 years); three (8.57%) were males and 32 (91.43%) were females. Data on maximum phonation time for the whole group and for females can be found on Table 1.

**Table 1:** Maximum phonation time n seconds for the group as a whole and female patients

		Total Group			Female Patients	
	MPT * preop	MPT 1-week postop	MPT 1-month postop	MPT preop	MPT 1-week postop	MPT 1-month postop
Mean	15,71	13,09	14,97	15,5	12,75	14,66
Median	16	14	15	15	14	15
Standard deviation	2,35	3,07	2,63	2,33	2,95	2,51
Minimum	11	4	7	11	4	7
Maximum	19	19	19	19	17	18
Total	35	35	35	32	32	32

* MPT: Maximum Phonation Time

Statistically significant differences for maximum phonation time among women were found between preoperative and postoperative tests done one week after surgery (p<.001) and postoperative tests done one week and thirty days after surgery (p<.001). Auditory perception test findings are shown in Table 2.

**Table 2:** Voice perception analysis – preoperative findings and results one week into postoperative care and 30 days after surgery

	Preoperative	Postoperative care one week after surgery	Postoperative care one month after surgery
**Degree of dysphonia**			
Normal	35	26	30
Altered	0	9	5
**Roughness**			
Normal	35	29	32
Altered	0	6	3
**Breathiness**			
Normal	35	32	33
Altered	0	3	2
**Weakness**			
Normal	35	33	33
Altered	0	2	2
**Stress**			
Normal	35	34	35
Altered	0	1	0

Preoperative videolaryngostroboscopy showed posterior glottal deviation after phonation of high pitch 'i' (as it sounds in Portuguese) in 5 (14.3%) patients and normal test results in 30 (85.7%) patients. Postoperative videolaryngostroboscopy performed one week after surgery showed laryngeal posterior deviation in eight patients, three of which seen only after surgery. No patients presented postoperative mobility disorders or vocal fold paralysis. Videolaryngostroboscopy performed a month after surgery revealed posterior laryngeal deviation in 6 patients; in five cases such finding had been identified preoperatively. The data on voice acoustic tests done preoperatively, one week after surgery, and 30 days after surgery are shown on Table 3.

**Table 3:** Voice acoustic evaluation parameters – whole group and female patients

		Whole Group			Female Patients	
	Preop	1-week Postop	1-month Postop	Preop	1-week Postop	1-month Postop
Fo (Hz)	226	211	210	227	213	212
Jitter%	0,612	0,7	0,57	0,61	0,71	0,6
Shimmer%	2,11	2,78	2,37	2,1	2,95	2,38
NHR	0,11	0,12	0,11	0,11	0,12	0,11

In regards to voice acoustic analysis performed along time, measurements of fundamental frequency showed statistically significant differences between results observed preoperatively and one week after surgery (p=0.0063), and preoperative results and values observed one month after surgery (p=0.0068). Fundamental frequency results one week and one month after surgery showed no statistically significant difference (p=0.6326). The injury-free group (n=32) also presented statistically significant differences in terms of fundamental frequency when preoperative and one-week postoperative (p=0.0144) and preoperative and one week post surgery results (p=0.0055) and one week and 30 days after surgery findings (p=0.0355) were compared. Harmonic noise ratio findings also showed statistically significant differences when preoperative and one week after surgery results were compared (p<0.0001) and one week post surgery and 30 days after surgery findings were compared (p=0.0005). The injury-free group also had statistically significant differences in relation to the same periods, respectively (p<0.0001) and (p=0.0012). The electromyography tests done one month after surgery revealed disorders in three patients. All patients diagnosed with superior laryngeal nerve external branch injury confirmed after cricothyroid EMG were positive for papilliferous carcinoma after FNA, a finding confirmed later through histopathology tests. All patients with posterior deviation (n=3) at this moment were diagnosed with superior laryngeal nerve external branch injury confirmed through EMG. No patients with benign disease were diagnosed by EMG with superior laryngeal nerve external branch injury. Nine (25.7%) patients had voice disorders one week after surgery. Such disorders were characterized mainly by mild hoarseness, vocal exhaustion, and difficulty to sing. Five (14.2%) patients remained with difficulty to sing or produce higher pitch sounds for over thirty days after surgery, although their voices were reported as normal.

## DISCUSSION

Thyroid surgery is traditionally performed on patients under general anesthesia, although historically the procedure has been conducted under local anesthesia. In 1907, Thomas Peel Dunhill reported on his experience with seven cases of thyrotoxicosis treated with thyroidectomy under regional anesthesia^5^. Within the last two decades there has been growing interest in this technique, stressing the preference given to patients and for performing the procedure in an outpatient setting with early discharge^1^, ^2^, ^3^. In our study we performed lateral traction of the sternothyroid and sternohyoid muscles without sectioning or detaching them, followed by selective ligation of the vessels in the superior thyroid pedicle after cricothyroid space dissection. The external branch of the superior laryngeal nerve was not dissected in any of the patients, except when it was naturally exposed after cricothyroid space dissection. The superior laryngeal nerve external branch was observed in 18 (51.4%) patients. Hisham et al.^6^ defined the cricothyroid space as the potentially non-vascularized space located medially in relation to the upper portion of the thyroid, and showed its relevance in reducing the incidence of injuries to the superior laryngeal nerve external branch after its dissection in thyroidectomy procedures. Selective ligation of the vessels in the superior thyroid pedicle in the area close to the upper portion of the thyroid gland has been described by some authors as a preventive measure against injuries to the external branch of the superior laryngeal nerve. Loré et al.^7^ reported on their experience with thyroidectomy procedures performed with selective ligation of the superior thyroid pedicle vessels close to the capsule of the thyroid gland. Although they used vocal arching observed under laryngoscopy as a sign of injury, 0.1% of their patients had definitive injuries to the superior laryngeal nerve external branch and 7.5% of them had permanent voice disorders. In our study, voice subjective analysis was performed followed by vocal assessment done in a lab setting (voice acoustic analysis), preoperative and postoperative videolaryngostroboscopy one week and 30 days after surgery, alongside cricothyroid muscle electromyography one week and thirty days after surgery. Significant reductions on maximum phonation time were observed in women when comparing preoperative and one week postoperative test results. Significant differences were also observed between findings gathered one week and thirty days after surgery. The same reduction was observed in males, but no statistical analysis was performed due to the limited number of male patients available in our study. Some authors have shown that the degree of dysphonia and roughness were significantly increased after thyroidectomy without evidence of vocal fold paralysis^8^,^9^. Voice perception analysis made evident that the one week postoperative degree of dysphonia and roughness was altered in 25.7% and 17.1% of patients respectively. Additionally, asthenia and stress levels were altered at this time only in patients with injured superior laryngeal nerve external branches confirmed via electromyography. Sataloff et al.^10^ verified the relevance of voice objective analysis in assessing patients with injured superior laryngeal nerve external branches. They also showed significant reduction on maximum phonation time in patients with superior laryngeal nerve unilateral palsy. In laryngeal nerve injury-free patients, Hong & Kim^11^ did not find significant differences for maximum phonation time and objective voice analysis after thyroidectomy. Our study observed statistically significant changes on maximum phonation time, fundamental frequency, percent shimmer, and harmonic noise ratio when comparing preoperative results and tests conducted one week and then one month after surgery, even in the group free of injury to the superior laryngeal nerve external branch confirmed under electromyography of the cricothyroid muscle. Although percent jitter presented changes when comparing pre and postoperative test results, no statistically significant differences were found in the group as a whole or within female patients. As also seen by other authors, dysphonia was more intense immediately after surgery^4^,^9^. Most patients had normal voices 30 days after surgery. Voice acoustic tests are limited when the purpose is to assess long term dysphonia, as many patients have altered vocal parameters but still present normal voices. More intense alterations in auditory perception tests and voice acoustic evaluations were observed in the group with injury than in the uninjured patient group. In an extensive literature review, Arnold^12^ described the main laryngeal alterations seen in superior laryngeal nerve as absence of accentuated vocal fold opening and closure, shortening of the vocal fold ipsilateral to the injury, vertical asymmetry between vocal folds, mucosal wave asymmetry, and glottal torsion with deviation of the posterior commissure towards the affected side. In our study, 14.3% of the patients had preoperative posterior glottal deviation. These patients had normal voice subjective and acoustic test results preoperatively. Dedo^13^ and Faaborg-Andersen^14^ described posterior glottal deviation in patients with uninjured superior laryngeal nerve external branch. All patients with superior laryngeal nerve external branch injury had posterior glottal deviation under postoperative videolaryngostroboscopy examination. Some authors reported posterior glottal deviation in patients with injured superior laryngeal nerve external branches^15^,^16^, while other confirmed such finding after electromyography^17^. It is important to state that apart from clinical and laboratory voice examination, our patients were also submitted to electromyography to assess them for possible injuries to the external branch of the superior laryngeal nerve as a cause for voice alteration or posterior glottal deviation. This is a relevant fact, as electromyography is considered t be the golden standard for the diagnosis of laryngeal nerve injuries. Janson et al.^18^ used electromyography and found 58% of temporary injuries to the superior laryngeal nerve external branch after thyroidectomy. Therefore, our study emphasizes the relevance of adopting an adequate method to assess the prevalence of voice and rima glottidis disorders after thyroidectomy.

## CONCLUSION

Voice disorders were observed immediately after surgery in 25.7% of our patients and in 14.2% of them later on. All patients underwent auditory perception and acoustic voice tests. Disorders were more pronounced in the group with injured superior laryngeal nerve external branches than in the group of dysphonic uninjured patients. Oblique glottis and posterior glottal deviation may be present in normal patients, but the onset of these conditions after thyroidectomy is indicative of injury to the superior laryngeal nerve external branch. Voice disorders or rima glottidis deviation may occur in normal patients. Therefore, these findings are non-specific to detect superior laryngeal nerve external branch injury.
